# Double perovskite cathodes for proton-conducting ceramic fuel cells: are they triple mixed ionic electronic conductors?

**DOI:** 10.1080/14686996.2017.1402661

**Published:** 2017-12-31

**Authors:** Helena Téllez Lozano, John Druce, Samuel J. Cooper, John A. Kilner

**Affiliations:** ^a^ Electrochemical Energy Conversion Research Division, International Institute for Carbon-Neutral Energy Research (I^2^CNER), Kyushu University, Fukuoka, Japan; ^b^ Dyson School of Design Engineering, Imperial College London, London, UK; ^c^ Department of Materials, Imperial College London, London, UK

**Keywords:** Proton conductors, cathodes, isotopic exchange, SIMS, mixed conductors, 50 Energy materials, 207 Fuel cells / Batteries / Super capacitors, 107 Glass and ceramic materials

## Abstract

^18^O and ^2^H diffusion has been investigated at a temperature of 300 °C in the double perovskite material PrBaCo_2_O_5+*δ*_ (PBCO) in flowing air containing 200 mbar of ^2^H_2_^16^O. Secondary ion mass spectrometry (SIMS) depth profiling of exchanged ceramics has shown PBCO still retains significant oxygen diffusivity (~1.3 × 10^−11^ cm^2^s^−1^) at this temperature and that the presence of water (^2^H_2_^16^O), gives rise to an enhancement of the surface exchange rate over that in pure oxygen by a factor of ~3. The ^2^H distribution, as inferred from the ^2^H_2_^16^O^−^ SIMS signal, shows an apparent depth profile which could be interpreted as ^2^H diffusion. However, examination of the 3-D distribution of the signal shows it to be nonhomogeneous and probably related to the presence of hydrated layers in the interior walls of pores and is not due to proton diffusion. This suggests that PBCO acts mainly as an oxygen ion mixed conductor when used in PCFC devices, although the presence of a small amount of protonic conductivity cannot be discounted in these materials.

## Graphical abstract

**Figure:**
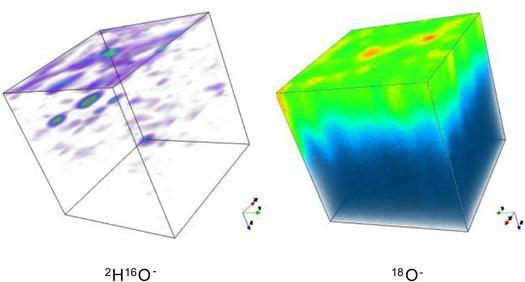

## 1. Introduction

Solid oxide fuel cells (SOFCs) for the direct conversion of chemical into electrical and heat energy are being commercialised in a number of countries to meet the strengthening emissions targets for CO_2_. Most of the development is concentrating on cells based on oxide ion conductors such as the fluorite-structured zirconia-based materials, which generally need temperatures of 800 °C or above to operate efficiently. The trend over the last decade has been to lower the temperature of operation towards the intermediate temperature regime (IT-SOFCs) of ≤650 °C to ameliorate the need for expensive high temperature materials and in order to control degradation rates to acceptable levels. One further attractive aspect of the SOFC is its ability to operate reversibly and consume spare electrical and heat energy for the electrolysis of steam, to generate hydrogen, as a component in a system for the storage of renewable primary energy such as wind or solar [1].

Amongst the candidate SOFC devices being investigated are those based on proton-conducting oxides, as these materials can give high conductivities at low temperatures a distinct advantage over the oxide ion based variant, whilst also being capable of operating in a reversible manner. One of the critical aspects of these proton conductor-based devices is the choice of the air electrode (i.e*.* the cathode for the fuel cell and the anode in the case of the electrolyser). This is difficult as the temperature of operation of these cells is limited, imposed by a decrease in proton concentration with increasing temperature [2,3] leading to a maximum in the protonic conductivity of the electrolyte materials [4] (see also the chapter by Norby in [5]). This temperature restriction imposes a need for materials that display a high activity for the oxygen reduction reaction (ORR) in the intermediate temperature regime. Many of the materials that have been investigated for this purpose are mixed ionic electronic conducting (MIEC) materials that have been demonstrated to function well as cathodes in SOFCs with oxide ion conducting electrolytes. Examples of these materials include the aliovalent substituted perovskite oxides (A_1-*x*_A′_*x*_BO_3-*δ*_, where A′ is a lower valent cation, usually an alkaline earth such as Sr or Ba), e.g*.* La_1-*x*_Sr_*x*_CoO_3-*δ*_ (LSC) [6]; double perovskite oxides (AA′B_2_O_5+*δ*_), e.g*.* GdBaCo_2_O_5+*δ*_ (GBCO) [7]; and the 214 Ruddelsden Popper oxides (A_2_BO_4+*δ*_), e.g*.* La_2_NiO_4+*δ*_ [8]. All these oxides are characterised by their high levels of mixed oxygen ion/electronic conductivity and high values of the surface exchange coefficient for oxygen, and have been dubbed as oxygen-MIECs (O-MIECs).

Recently, there have been a number of publications that show that the same materials should function well as cathodes in proton-conducting ceramic fuel cells (PCFCs) [9–18]. This has led to interest in the manner in which these materials operate as cathodes in these protonic conducting cells. For example, Mauvy and co-workers have a number of publications in which they propose that the cathode materials contain significant protonic conductivity and thus the mode of operation of these materials is as a mixed conducting material that can exhibit, electronic (p-type), oxygen ion and protonic conductivity (P-MIEC) [9–13]. This suggestion seems logical because proton conductivity is observed in the 2,4 perovskites, such as substituted barium cerate and zirconate. More recently Zohourian et al. have suggested that a protonic conductivity of ~10^−5^ S cm^−1^ could be sufficient to make significant parts of the electrode surface active [19].

Direct observation of protonic species in these mixed conductors is rather sparse. Han et al. [20] have used the Karl Fischer titration method to determine the proton concentration in the solid solutions La_1−x_Sr_x_Sc_1−y_Fe_y_O_3−δ_ (LSSF) exposed to water vapour at 300 °C. They found that the proton concentration dropped to low levels with the addition of Fe and conclude that LSSF is probably not a mixed protonic electronic conductor. They also measured the protonic concentrations in LSC and La_0.6_Sr_0.4_Co_1−y_Fe_y_O_3−δ_ (LSCF) under similar conditions and concluded that, again, there was a low proton concentration and this would give rise to negligible protonic conductivity.

### 1.1. Defect chemistry

If we take the example of barium zirconate substituted with a lower valent cation such as yttrium on the B-site we have the following possible substitution reaction.

(1) $${\text{Y}}_{2}{\text{O}}_{3}\to 2{\text{Y}}_{\text{Zr}}^{\prime }+3{\text{O}}_{\text{O}}^{\text{x}}+{\text{V}}_{\text{O}}^{\cdot \cdot }$$

Followed by the reaction of the oxygen vacancies with water to produce protonic species

(2) $${\text{V}}_{\text{O}}^{\cdot \cdot }+{\text{O}}_{\text{O}}^{\text{x}}+{\text{H}}_{2}\text{O}↔2{\text{OH}}_{\text{O}}^{\cdot }$$

There is also the competing reaction of the oxidation of the vacancies to produce electron holes.

(3) $${\text{V}}_{\text{O}}^{\cdot \cdot }+\frac{1}{2}{\text{O}}_{2}↔2{\text{h}}^{\cdot }+{\text{O}}_{\text{O}}^{\text{x}}$$

The protonic species can also be produced at lower oxygen pressures by a further reaction consuming electronic holes, dubbed hydrogenation by Poetzsch et al*.* [21]

(4) $$2{\text{h}}^{\cdot }+2{\text{O}}_{\text{O}}^{\text{x}}+{\text{H}}_{2}\text{O}↔2{\text{OH}}_{\text{O}}^{\cdot }+\frac{1}{2}{\text{O}}_{2}$$

From Equations (1–4) the neutrality condition is.

(5) $$\left[{\text{Y}}_{\text{Zr}}^{\prime }\right]=\text{p}+\left[{\text{OH}}_{\text{O}}^{\cdot }\right]+2\left[{\text{V}}_{\text{O}}^{\cdot \cdot }\right]$$

The effective negative charge introduced by the aliovalent substitutional is balanced by three charged species, all of which could be mobile. There is nothing specific to the host oxide, or substitution site, in Equations (2–4) thus any oxide substituted with lower valent cations can have populations of the three possible charge carriers. What matters for the mass and electrical transport properties of these materials is the relative concentrations of the three charge compensating species and their mobility under cell operating conditions. The concentration of compensating defects will depend upon the reaction constants for the reactions given in Equations (2–4). In addition, it must be remembered that the substitutional, e.g*.* ${\text{Y}}_{\text{Zr}}^{{}^{\prime }}$ can be compensated by any species with an effective positive charge e.g*.* anion contaminants. It has been shown some time ago that anionic substitution e.g*.* ${\text{F}}_{\text{O}}^{\cdot }$ is also possible in these perovskites [22], although it is not clear that this type of substitution will result in a mobile species.

Most of the MIEC materials used as SOFC cathodes are a little different to the materials mentioned above as they are based on the 3,3 perovskites. They are also substituted, but usually on the A-site of the ABO_3_ perovskite. An example is the set of materials based upon the general formula La_1-*x*_Sr_*x*_CoO_3-*δ*_. We should be aware that the same neutrality condition for the aliovalent substitution applies for these 3,3 perovskites, as stated in Equation (6) below, thus the possibility of significant proton concentrations also exist for these MIEC materials and evidence for this has been sought experimentally.

(6) $$\left[{\text{Sr}}_{\text{La}}^{\prime }\right]=\text{p}+\left[{\text{OH}}_{\text{O}}^{\cdot }\right]+2\left[{\text{V}}_{\text{O}}^{\cdot \cdot }\right]$$

### 1.2. Electrode reactions

**Figure 1.**
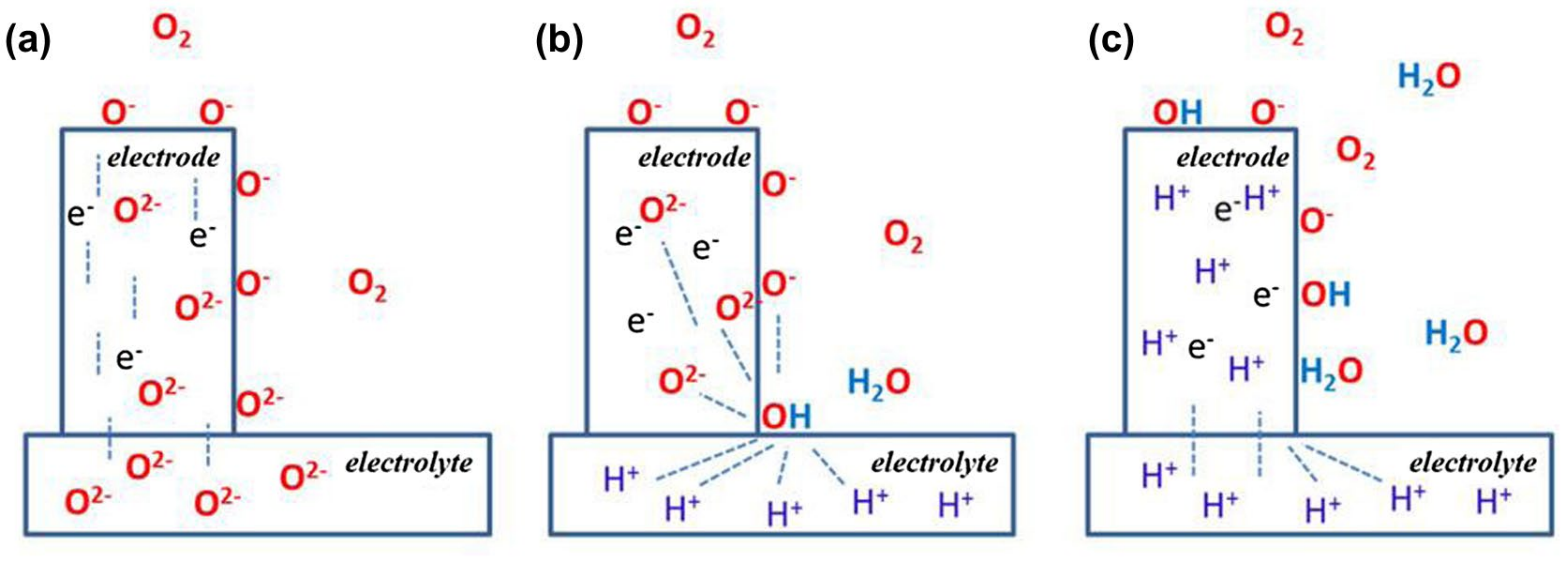
Illustration of the oxygen electrode mechanisms in an O-MIEC electrode material on (a) an oxide ion conductor and (b) a proton conductor, as well as (c) a P-MIEC electrode material on a proton conducting electrolyte. Reprinted with permission from [23], © Elsevier 2015.

Much of the evidence for the existence of significant concentrations of protonic species in these cathode materials comes from electrochemical studies and thermogravimetric [23] experiments in wet atmospheres. Based on these observations Strandbakke [23] and others have proposed two models for the operation of these cathodes with protonic electrolytes as distinct for the same materials in an SOFC with an oxide electrolyte. These models are shown in Figure 1.

In the case where there is no protonic conductivity in the MIEC, but there is sufficient oxygen ion conductivity (Figure 1(b)), then the electrode reaction in a PCFC will be split into a reaction at the triple phase boundary (tpb) given by;

(7) $${\text{O}}_{\text{O}(\text{electrode})}^{\text{x}}+2{\text{OH}}_{\text{O}(\text{electrolyte})}^{\cdot }↔{\text{H}}_{2}\text{O}+{\;\text{V}}_{\text{O}\left(\text{electrode}\right)}^{\cdot \cdot }+2{\text{O}}_{\text{O}(\text{electrolyte})}^{\text{x}}$$

with the accompanying reaction on the surface of the MIEC electrode

(8) $$2{\text{e}}^{-}+\frac{1}{2}{\text{O}}_{2}+{\text{V}}_{\text{O}}^{\cdot \cdot }↔{\text{O}}_{\text{O}}^{\text{x}}$$

If the cathode material has sufficient protonic conductivity then the desired electrode reaction at the surface of a P-MIEC in a PCFC (Figure 1(c)) would be given by;

(9) $$2{\text{e}}^{-}+\frac{1}{2}{\text{O}}_{2}+2{\text{OH}}_{\text{O}}^{\cdot }↔2{\text{O}}_{\text{O}}^{\text{x}}+{\text{H}}_{2}\text{O}$$

In contrast to the reaction for an O-MIEC in a SOFC (Figure 1(a))

(10) $$2{\text{e}}^{-}+\frac{1}{2}{\text{O}}_{2}+{\text{V}}_{\text{O}}^{\cdot \cdot }↔{\text{O}}_{\text{O}}^{\text{x}}$$

The advantage of having P-MIEC behaviour as embodied in Equation (9) is a much larger part of the surface of the P-MIEC would be active for the electrode reaction, rather than being limited to the tpb. It would be the direct analogue of using an O-MIEC as an SOFC cathode where the cathode surface has been shown to be active in the ORR [24].

Clearly the dominant mechanism for a given electrode system will depend upon the majority ionic carrier in these MIEC oxides (H^+^ or O^2−^) but there is little relevant data in the literature to distinguish between the models shown in Figure 1(b) and (c) and detailed in Equations (7–9).

### 1.3. Double perovskites

Some of the best performing materials as PCFC cathodes are those with a layered double perovskite structure [9,14,17,23,25]. These double perovskites are a sub group of the A_1-*x*_A′_*x*_BO_3-*δ*_ materials mentioned above. For compositions where *x* = 0.5 the A and A′ cations can order to give a layered double perovskite structure, often written as AA’B_2_O_5+*δ*_, and the oxygen vacancies can order at low temperatures. This can modify the schema given in Equations 1–6 but the same general principles hold. Density functional theory calculations [26] have indicated that the double perovskite GBCO might support a population of protonic species. A neutron powder diffraction study of the system NdBaCo_2−*x*_Mn_*x*_O_5+*δ*_ (*x* = 0 and 0.5) in wet (D_2_O) atmospheres found little evidence of proton uptake [27] for either of the compositions studied, but a ready loss and re-incorporation of oxygen. For the most promising group of these potential cathode materials, the double perovskites, there are many reports of the diffusivity of the oxygen [28–36], but only one report of surface exchange of ^2^H has been made in NdBaCo_2_O_5+*δ*_ (NBCO) and this has not been widely disseminated [37]. Even here, the diffusion coefficient reported at 260 °C is ~10^−15^ cm^2^s^−1^, which is low compared to the extrapolated oxygen diffusion coefficient for this type of material (~10^−12^ cm^2^s^−1^) [32]. Thus, there is a need for the systematic and direct study of the transport properties of the materials in question, to support inferences made from indirect measurements such as electrochemical and thermogravimetric methods.

**Figure 2.**
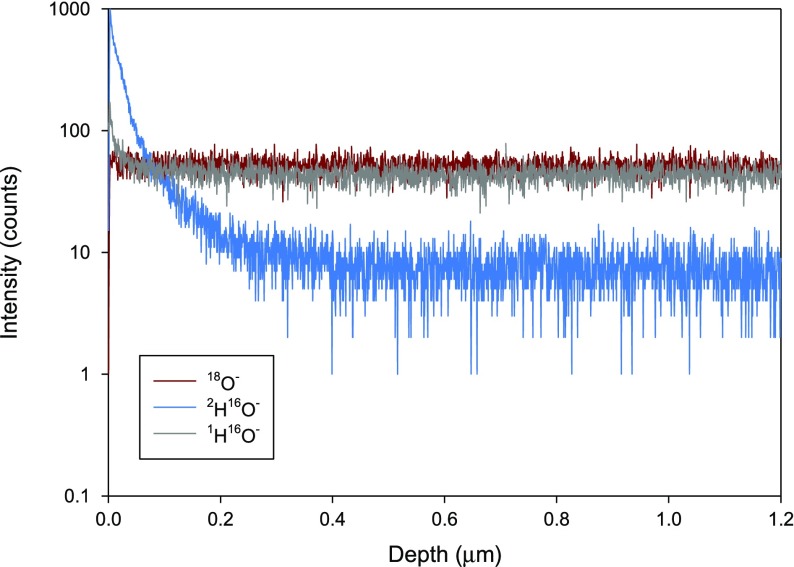
Depth profile of an as prepared GBCO sample after annealing in D_2_O at 300 °C for 6 h. Reprinted with permission from [38], *©* European Fuel Cell Forum (EFCF).

The aim of the present experiments is to follow on from an earlier investigation by Sharp et al. [38] into the degree of protonation of sintered and dense GBCO samples annealed in H_2_O bearing environments. In these experiments samples with two different pre-treatments were subjected to a set of anneals at 300 or 400 °C, first in an atmosphere of pure water at a pressure of 70 mbar, to introduce an equilibrium concentration of protons into the material, followed by a much shorter anneal in ^2^H_2_^16^O (D_2_O) at the same pressure to determine a ^2^H isotopic diffusion coefficient. The pre-treatments involved either leaving the sample in the as-polished state or having a high temperature pre-anneal at 950 °C in pure oxygen at 200 mbar, immediately followed by the exposure to natural water at the chosen anneal temperature. The rationale behind this choice of pre-anneal was to remove any pre-existing surface reaction products that might form on exposure of the as-prepared sample to the ambient atmosphere, such as hydroxides, carbonates or sulphates. When depth profiled using time-of-flight secondary ion mass spectrometry (ToF–SIMS) at sufficient mass resolution to distinguish between ^18^O^−^ and ^2^H^16^O^−^, the sample without a pre-anneal gave the apparent diffusion profile shown in Figure 2, yielding a diffusion coefficient of 4.5 × 10^−15^ cm^2^s^−1^. This is comparable to the value for NBCO at 260 °C reported by Goupil [37], who used a slightly different technique omitting the first anneal in natural water and thus measured an apparent chemical diffusion coefficient.

In a second sample, which was pre-annealed in pure oxygen and not exposed to the ambient atmosphere, the profile of ^2^H^16^O^−^ was ***completely absent***, and only the instrumental background level of this signal was observed. This implied that the apparent deuteron penetration profile seen in the first sample was associated with the presence of a corrosion layer on the outer surface of the GBCO which interacts with water to give rise to a hydrated surface layer. Although the samples were processed to a high density, the presence of a few residual pores coated with this hydrated layer in the analysed area, could give rise to an apparent ^2^H penetration profile seen in Figure 2.

One comment must be made at this point, and that refers to the conditions under which the water is introduced to the samples. In both sets of experiments reported in [37,38] and the water was introduced in closed systems without the presence of ambient air. This is not the operating environment of a working cathode where moisture is a component of the ambient. In order to determine the relative importance of oxygen transport and proton transport in a more realistic cathode ambient, and to provide some evidence that should discriminate between the two cathode mechanisms for protonic cells, mentioned earlier, we chose to study the related mixed conducting PrBaCo_2_O_5+*δ*_ (PBCO) employing the new method of back-exchange [39] to simultaneously explore both the protonic and oxygen exchange processes.

## 2. Experimental details

### 2.1. Sample synthesis

PBCO powder was prepared by solid state synthesis from high purity precursors. Stoichiometric amounts of dried Pr_6_O_11_ (Purity >99.9%, Wako Pure Chemical Industries, Ltd., Osaka, Japan), Co_3_O_4_ (Purity >99.9%, Sigma Aldrich, Tokyo, Japan) and BaCO_3_ (Purity >99.9%, Wako Pure Chemical Industries, Ltd.) were mixed and ground with a mortar and pestle before calcination at 1000 °C for 12 h. X-ray diffraction analysis was performed in Bragg–Brentano geometry (Rigaku X-ray diffractometer, RINT 2500 HLR, Rigaku Co., Tokyo, Japan) confirming the phase purity of the calcined powder (indexed as an orthorhombic structure, PDF-00-056-0366) with no detectable secondary phases. The calcined powders were pressed into pellets by uniaxial pressing, followed by isostatic pressing at 300 MPa for 30 min) and sintering at 1200 °C for 12 h. The density of the sintered pellets was measured by the Archimedes method to be 99% of theoretical, indicating the samples were gas tight and therefore suitable for ^18^O_2_ and ^2^H_2_^16^O exchange experiments). The pellets were polished successively using a diamond suspension to a final mirror-like surface, where the last step was performed by using a ¼ μm-particle size suspension. In order to remove any residue from the polishing process, the samples were cleaned successively in an ultrasonic bath using ultrapure acetone, ethanol and deionised water. The sample microstructure showed several large cracks due to the high chemical expansion coefficient of PBCO and very fine distributed porosity. The grain size is on the order of 5 μm.

### 2.2. Sample isotopic exchange

Previous work on GBCO involved isotopic exchange studies (^16^O/^18^O and ^1^H/^2^H) in dry and wet atmospheres of samples using the isotope exchange depth profile method (IEDP) [38]. This formed the basis for the modified experimental technique used in this study, however there were several modifications made to allow the use of humidified air as the exchanging gas, as well as the simultaneous measurement of ^18^O and ^2^H^16^O exchange.

One way to do this is to use doubly labelled water, i.e*.* ^2^H_2_^18^O, in the exchanging gas, however this is a very expensive option and in order to circumvent this problem a double exchange technique, known as back-exchange, was used [39]. In this method, the first exchange is made in dry ^18^O_2_ to introduce a diffusion profile, which can be measured at this stage by ToF-SIMS. This is followed by a second exchange in any ambient containing natural oxygen (i.e*.* ^16^O) bearing molecules such as H_2_O, CO_2_, SO_2_ etc. with the proviso that the *P*o_2_ is maintained constant for the two anneals. The existing ^18^O profile is then altered by two processes, a broadening of the distribution due to thermally activated diffusion, and the exchange and diffusion of the ^16^O in the gas phase with the ^18^O in the solid phase. This produces a distribution with a shallow peak which can be fitted using a numerical method to yield an oxygen diffusion coefficient for each anneal, as well as separate surface exchange coefficients for the first dry exchange and the second exchange in the chosen ambient.

**Figure 3.**
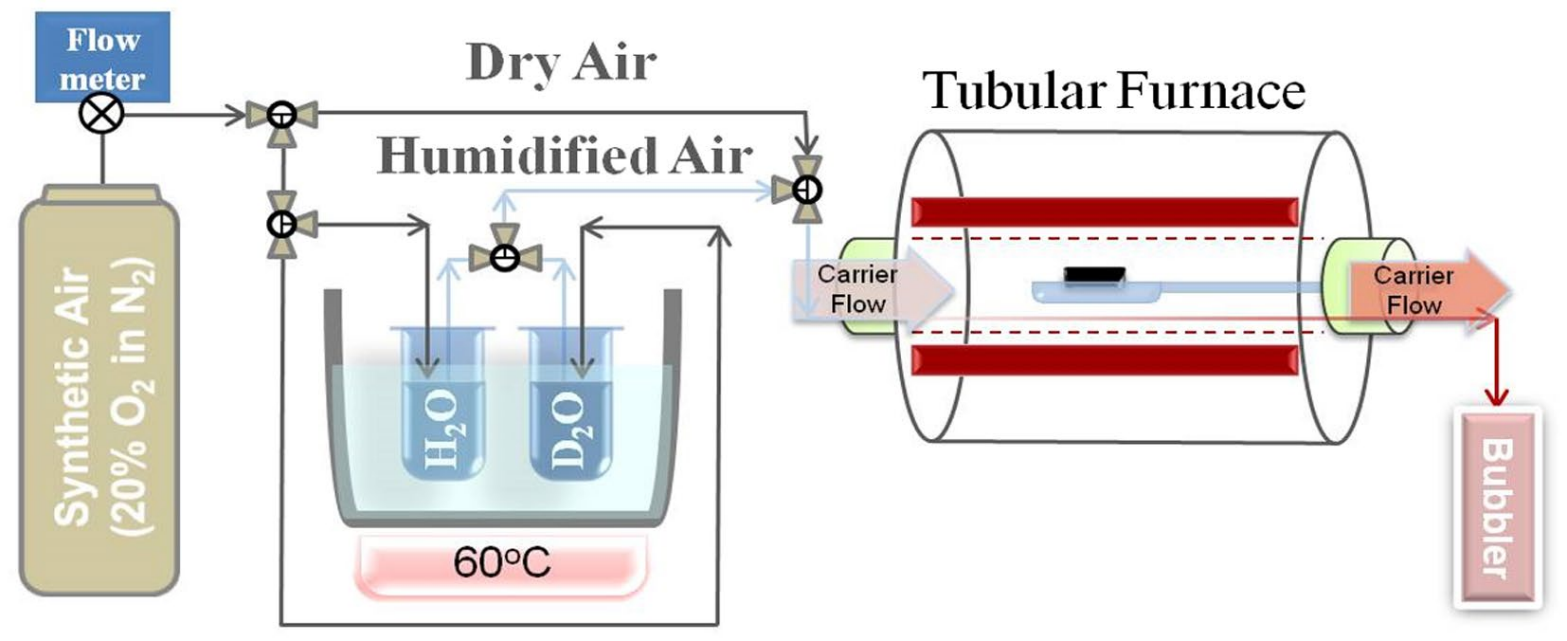
Schematic of apparatus used to anneal samples in humidified ambient.

The PBCO samples were given the first anneal in dry ^18^O_2_ exchanged in a custom-built apparatus which could be evacuated to ultra high vacuum to minimise the amount of H_2_O in the annealing tube, before backfilling with either high purity natural oxygen or oxygen enriched in ^18^O to an isotopic fraction of 0.9 for the annealing. For the experiments using humidified atmospheres, the samples were placed in a silica boat in the centre of an alumina tube furnace through which a carrier gas of bottled high purity synthetic air (20 vol.% O_2_ in N_2_) could be passed at a controlled flow rate (Figure 3). The gas could be passed through a bubbler containing water enriched with ^2^H_2_O to an isotopic fraction of 0.99 placed in a constant temperature water bath set at 60 °C giving a saturated water vapour pressure of 199.5 mbar. To prevent any condensation of the water, all the interconnecting stainless steel pipework was heated to temperatures in excess of 100 °C by insulated heating tape.

Two different sample preparation routes were taken to provide samples for SIMS analysis. This is detailed in Table 1 below.

**Table 1.** Summary of treatment given to samples prior to SIMS analysis.

Sample no.	Equilibration anneal in dry ^16^O_2_ at 300 °C/hours	Exchange anneal in dry ^18^O_2_ at 300 °C/hours	Back-exchange anneal in flowing synthetic air with 200 mbar ^2^H_2_^16^O at 300 °C/hours
1	20	2	
2	20	1.5	0.5

### 2.3. ToF-SIMS analyses

The samples were analysed by ToF-SIMS using a ToF-SIMS 5 instrument (ION-TOF GmbH, Münster, Germany) equipped with a ‘Nanoprobe’ liquid metal ion gun (LMIG) producing Bi^+^ primary ions at 30 keV. The LMIG was operated in the high current mode with bunched primary ion pulses, to afford the best mass resolution. Depth profiling was performed by combining cycles of analysis by the primary beam with a separate 2 keV Ar^+^ sputter beam at 45° incidence. The Ar^+^ beam was used to sputter a 250 × 250 μm crater, with the central region 150 × 150 μm analysed by the Bi^+^ primary beam. Care was taken to avoid regions close to prominent cracks on the sample surface which could affect the measure diffusion profiles.

The ‘reflectron’ time-of-flight analyser was operated in a high mass resolution mode, involving reducing the ion energy in the drift tube to 1 keV. To avoid saturation of the detector by the intense signal for ^16^O^−^, whilst maintaining sensitivity for other weaker peaks (particularly ^18^O^−^ and ^2^HO^−^), selective attenuation of secondary ions (SASI) [40] was applied to the ^16^O^−^ peak.

The sputter depth profiles were depth calibrated by measuring the depth of the sputter crater after the SIMS analysis using an OLS 4000 3D laser microscope (Olympus, Japan), and assuming a constant sputter rate throughout the profile.

## 3. Results

**Figure 4.**
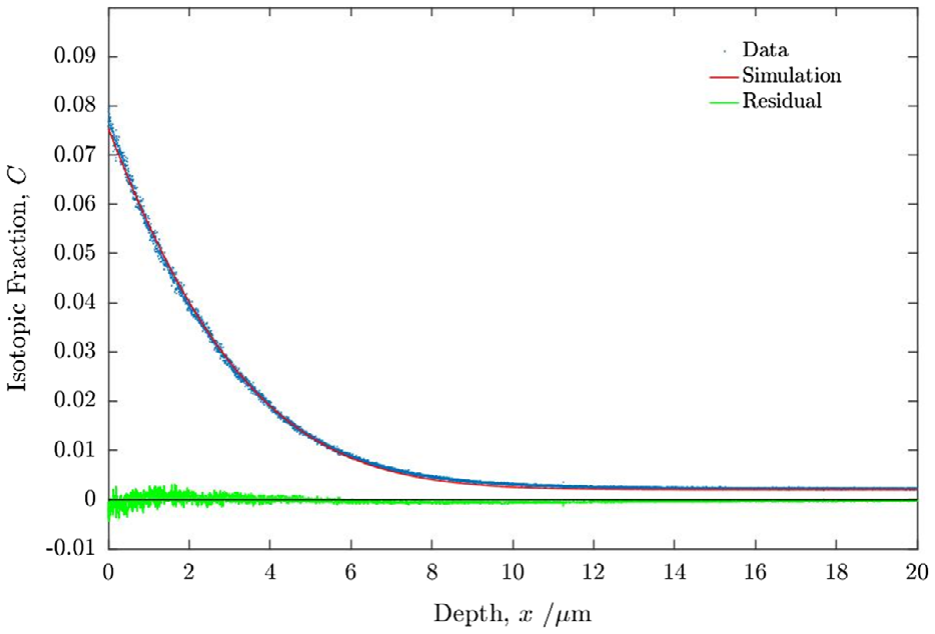
Oxygen isotopic (^18^O) exchange depth profiles for PBCO sample 1 measured by ToF-SIMS. For details of the anneals and fitted coefficients see text.

The first stage of the process was to introduce an ^18^O diffusion profile into the PBCO sample (Sample 1) by sequentially annealing in high purity natural and then in isotopically enriched oxygen, under the conditions defined in Table 1. The resulting diffusion profile, shown in Figure 4, was obtained by ToF-SIMS depth profiling. Oxygen self-diffusion (*D* ^*^) and surface exchange (*k* ^*^) coefficients are estimated by fitting the measured profile to the well-known solution to the diffusion equation as described by Crank [41], and given below in Equation (11).

(11) $$C^{\prime }\left(t,x\right)=\frac{C\left(t,x\right)-C_{\text{bg}}}{C_{\text{gas}}-C_{\text{bg}}}=\text{erfc}\left(\frac{x}{2\sqrt{D^{*}t}\;}\right)-\exp\left(\frac{k^{*}x}{D^{*}}+\frac{k^{*2}t}{D^{*}}\right)\text{erfc}\left(\frac{x}{2\sqrt{D^{*}t}}+k^{*}\sqrt{\frac{t}{D^{*}}}\;\right)\;$$

The quantity $C^{\prime }\left(t,x\right)$ is defined as the isotopic fraction, *C*(*t*, *x*), at a specific time and depth, *t* and *x*, respectively, normalised to the background ($C_{\text{bg}}$) and gas phase ($C_{\text{g}}$) isotopic fractions. The solution ignores diffusion in the gas phase, by assuming it to be infinitely fast.

In Figure 4 the experimental data from the SIMS depth profile are shown together with the fitted profile and the residuals from the fit made using the open source MATLAB application TraceX [42]. The forward exchange data fit very well to the classical solution to the diffusion equation and the fitted diffusion parameters are listed in Table 2. The forward exchange data are entirely compatible with previous data for the diffusion and exchange parameters obtained for PBCO in dry oxygen [32].

**Table 2.** Fitted oxygen exchange parameters.

Sample no.	$D_{1}^{*}$/cm^2^s^−1^	$D_{2}^{*}$/cm^2^s^−1^	First exchange dry O_2_, $k_{1}^{*}$/cm s^−1^	Second exchange synthetic air plus ^2^H_2_^16^O, $k_{2}^{*}$/cm s^−1^
1	1.43 × 10^−11^	–	3.48 × 10^−9^	–
2	1.27 × 10^−11^	–	3.48 × 10^−9^	9.8 × 10^−9^
2	1.70 × 10^−11^	0.83 × 10^−11^	3.49 × 10^−9^	10.8 × 10^−9^

The next step in the process was to examine a sample exposed to the treatment labelled as sample 2 in Table 1. As mentioned above the diffusion parameters are now obtained using a finite difference method, which can be set to have four independent variables; $D_{1}^{*}$, $k_{1}^{*}$, $D_{2}^{*}$ and $k_{2}^{*}$ where the subscripts 1 and 2 refer to the first and second exchanges, respectively. Alternatively, a three-parameter fit can be obtained by setting $D_{1}^{*}=D_{2}^{*}$_._

**Figure 5.**
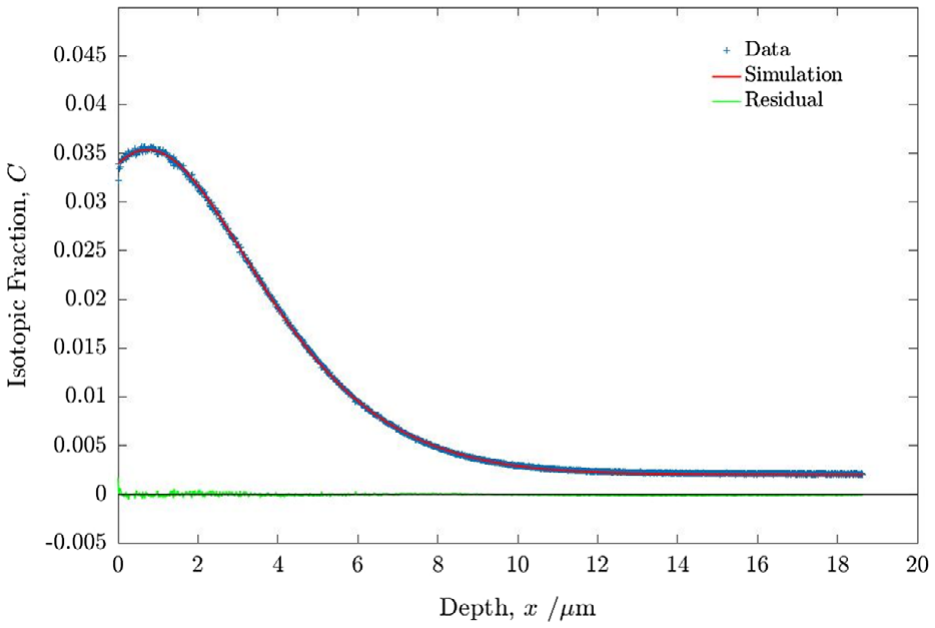
Oxygen isotopic (^18^O) depth profiles for PBCO sample 2 measured by ToF-SIMS. For details of the anneals and fitted coefficients see text.

Sample 2 was again depth profiled using the ToF-SIMS and the resulting depth profile fitted by the TraceX application. It was decided to compare both three and four variable fits to ensure the consistency of the fitting procedure and both are given in Table 2. The three variable fit produced a good fit to the experimental profile as can be seen in Figure 5. An increase in the value of the surface exchange coefficient, $k_{2}^{*}$, is observed where $k_{2}^{*}$ is almost three times larger than the value reported in pure oxygen. By allowing a greater degree of freedom in the four variable fit some differences between $D_{1}^{*}$ and $D_{2}^{*}$ were observed, with $D_{1}^{*}\geq D_{2}^{*}$, which probably reflects a slight difference in temperature of the two anneals, done in different furnaces one under a static atmosphere and the other under a flowing gas atmosphere. Despite this small difference the enhancement of $k_{2}^{*}$ by a factor ~ 3 still results. This observation was repeatable across a number of exchanged samples and suggests the water (D_2_O) molecule exchanges in preference to the dioxygen molecule and accelerates the measured surface exchange coefficient. Similar observations have been made for ceria and zirconia [43–45] solid solutions and more recently for other MIEC materials [46].

**Figure 6.**
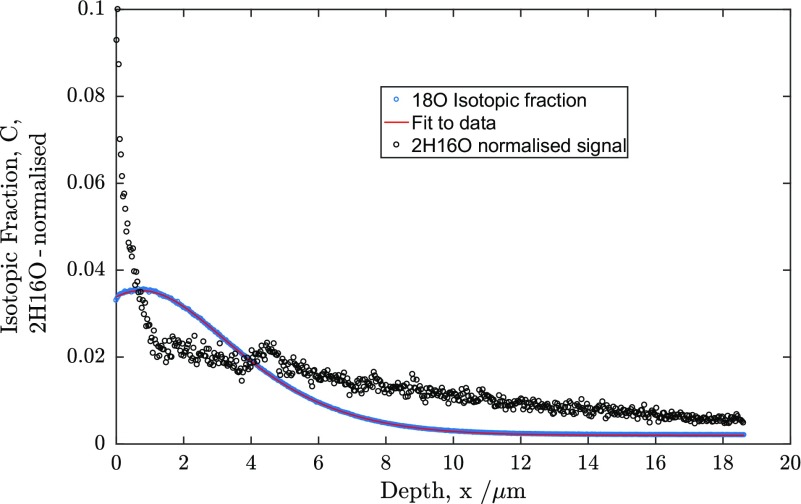
Comparison of the ^2^H^16^O^−^ signal with the isotopic exchange profile for sample 2.

Having established that the PBCO is still a good oxygen ion conductor (and hence O-MIEC) at these low temperatures, and has an enhanced rate of surface oxygen exchange, the question of the nature of the diffusion of any protonic species remains. In one of the series of experiments performed by Sharp et al*.* [38], a pre-treatment was given before exposure to moisture, and atmospheric exposure was avoided. This was not possible in these experiments and the sample was directly exposed to the atmosphere prior to the back-exchange in ^2^H_2_^16^O. This is similar to the experiments performed by Goupil [37] and would result in the development of a diffusion profile due to the chemical diffusion of the protons. By using the high mass resolution mode, the separation of the signals at mass 18 due to ^18^O^−^ and ^2^H^16^O^−^ was possible and a depth profile for the ^2^H^16^O^−^ was extracted. This is shown in Figure 6.

In order to follow the penetration profile of the ^2^H, the signal of ^2^H^16^O^−^ was extracted from the raw data and normalised for plotting on the same scale as the back-exchange profile. This is also shown in Figure 6. This profile consists of two parts, a sharp decrease at the surface down to ~1 micron and a longer tail into the material. It is not currently possible to fit this two-part profile to obtain a diffusion coefficient for the deuterons. If we assume that the shallow portion is due to bulk diffusion, then we can estimate a diffusivity of the deuterons of 1.4 × 10^−12^ cm^2^s^−1^. This is still an order of magnitude lower than the oxygen diffusivity but higher than found in earlier measurements [37,38].

**Figure 7.**
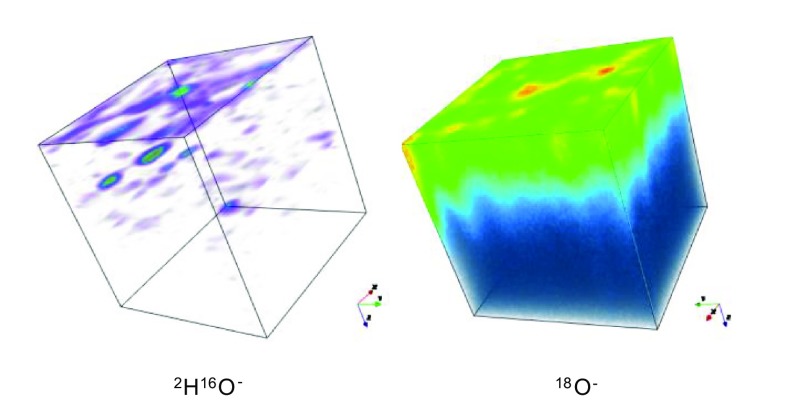
False colour 3D maps of the ^2^H^16^O^−^ and ^18^O^−^ ion intensities in the central 150 × 150 microns of the analysed area for sample 2.

The assumption in making this estimation is that the penetration of the ^2^H species is caused by isotropic diffusion in a semi-infinite medium. That this assumption is not valid is illustrated in Figure 7, where the 3D distributions of the ion intensities in the analysed volume have been shown using a false colour mapping. The ^18^O^−^ intensity shows a mostly homogeneous distribution with some high intensity regions, possibly pores that can be excluded when selecting areas to extract the isotopic depth profile, using the post analysis data processing software. This corresponds to an approximately planar diffusion front that would be expected, given the experimental protocol. In contrast the ^2^H^16^O^−^ distribution is much more heterogeneous and is dominated by ‘hotspots’ of intensity. Again, these would seem to be associated with large-scale defects in the ceramic materials such as pores.

## 4. Discussion

**Figure 8.**
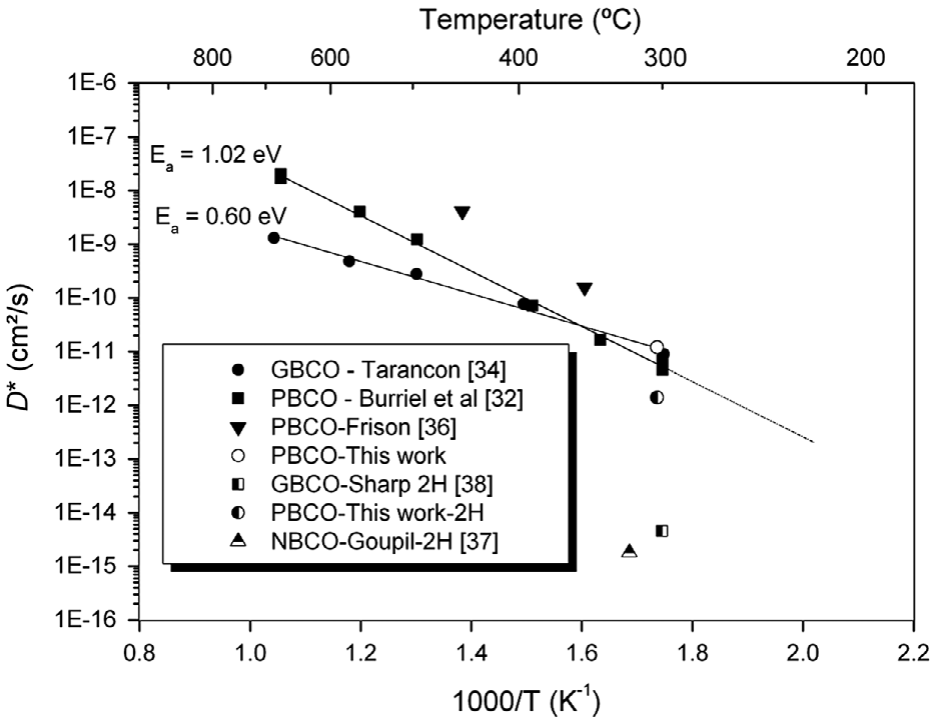
Comparison of literature data for oxygen and ^2^H diffusion coefficients and data from these experiments.

In order to assess the data obtained by this investigation it is perhaps worthwhile to first make a comparison of the oxygen diffusion data obtained in these experiments to previous published data on double perovskite materials, as shown in Figure 8. The data point obtained here at 300 °C is in good agreement with data previously published by Burriel et al. [32] and thus confirms that even at temperatures as low as 300 °C PBCO still retains significant oxygen diffusivity (i.e*.* it retains appreciable O-MIEC behaviour). Figure 8 also shows the data for ^2^H diffusion using the data for NBCO [37] and GBCO [38] and the estimated value for PBCO determined here. It is noticeable that despite the good agreement of the oxygen diffusion data there is considerable scatter in the ^2^H diffusion data from the three related compounds.

It is important to stress that these ^2^H data represents ‘apparent’ diffusion profiles and both the work of Sharp et al. [38] and this current work throws doubt on the fact that this is caused by bulk diffusion. As is discussed below, it is more likely to be caused by treatments at elevated temperatures leading to the presence of surface segregated layers of reactive elements which on exposure to ambient air can then become hydrated (and hence deuterated).

There is now a large body of evidence to show that these perovskite and perovskite-related materials will terminate with an AO or A′O surface after exposure to elevated temperatures in ambients with a high Po_2_ (~200 mbar) [28,47–54]. In particular for the Ba containing double perovskites, Ba has been shown to segregate to the outer surface after treatment at temperatures as low as 400 °C [28]. This is particularly relevant for sintered pellets, calcined powders and polished and annealed pellets that have undergone a heat treatment in such an oxygen rich ambient. Thus, most materials used in a wide range of experiments will have Ba rich surfaces which should be reactive to water and to the acidic components of the ambient atmosphere. Recent work on the related single perovskite O-MIEC material Ba_0.5_Sr_0.5_Co_0.8_Fe_0.2_O_3−δ_ by Berenov and co-workers [55] has shown that the surface carbonate and sulphate can form after exposure to air, confirming earlier work by Bucher et al*.* [56].

These BaO rich surfaces have been shown to be sensitive to the presence of water at elevated temperatures. Two papers by Slodczyk et al. [57,58] show that the electrolyte material BaCe_0.5_Zr_0.3_Y_0.16_Zn_0.04_O_3−*δ*_, when exposed in CO_2_ and H_2_O environments, have surface reactions causing the formation of BaCO_3_ and Ba(OH)_2_.*n*H_2_O, even at temperatures as low as 400 °C in 60 mbar of H_2_O. This was particularly noticeable to a sample exposed for 12 h. The sample had been cut and polished, prior to exposure, and the grain boundaries, visible on the polished surface, were preferentially attacked. This is particularly interesting as the fresh surface would not have been subject to strong Ba segregation, however the grain boundaries that have been exposed to the sintering temperature should be strongly Ba rich. The same argument applies to the internal surfaces of pores in sintered bodies that have been cut and polished for experiments such as oxygen exchange experiments described in this paper. It is thus probable that these Ba rich pore surfaces and exposed grain boundaries form hydrated layers on exposure to water vapour, especially at low temperatures, that can give erroneous results in these isotopic exchange experiments. Similar comments can be made about thermogravimetric analysis (TGA) experiments if the powders are subjected to pre-anneals at high temperatures before TGA experiments in a water bearing ambient. Powders with a high surface area and Ba-enriched surfaces should show a high affinity for water. TGA experiments on sintered samples such as those reported by Duan et al. on BaCo_0.4_Fe_0.4_Zr_0.1_Y_0.1_O_3-δ_ [59] and by Poetzsch et al. [60] on Ba_0.5_Sr_0.5_Fe_0.8_Zn_0.2_O_3-δ_ are more reliable and could indicate some protonic uptake into the bulk (or perhaps grain boundaries) of these materials.

To recap, the attempts to measure protonic diffusivity in three of these double perovskite materials NBCO [37], GBCO [38] and PBCO have either returned diffusivities that are 3–4 orders lower than that of the oxide ion [37,38] or diffusion profiles and distributions that are inconsistent with bulk proton diffusion, as shown here, or even absent when air exposure is excluded [38]. Thus, direct evidence for significant concentrations of protons with high mobility in these double perovskites has not been found. Returning to the electrode reactions in Equations (7–10), it looks apparent from the arguments given above that, in the double perovskite materials considered in the current work, the electrode reaction is dominated by Equations (7) and (8) i.e. the material is predominantly acting as an O-MIEC type cathode.

## 5. Conclusions

^18^O and ^2^H diffusion has been simultaneously investigated in the double perovskite material PBCO for the first time by using the back-exchange technique in flowing air containing a saturated vapour of ^2^H_2_^16^O. The back-exchange method has shown that PBCO still retains O-MIEC behaviour at low temperatures of 300 °C and that the presence of 200 mbar of water (^2^H_2_^16^O), gives rise to an enhancement of the surface exchange rate over that in pure oxygen by a factor of ~3. More interestingly the ^2^H distribution, as inferred from the ^2^H_2_^16^O^−^ signal, shows an apparent depth profile which could be interpreted as ^2^H diffusion. However, examination of the 3-D distribution of the signal shows it to be nonhomogeneous and probably related to the presence of large-scale defects such as pores. Discussion of the segregation effects in these materials and the likely effects they have upon exposure to moisture reinforces the idea that the distribution of ^2^H seen in these experiments are likely to have been caused by the presence of hydrated Ba compounds in the pores of the PBCO. It is likely that these findings have a broader application to materials that have been proposed as triple MIECS as they mostly contain basic cations such as the alkaline or rare earths. Returning to the electrode reactions, the evidence presented here strongly suggests that PBCO and GBCO act as O-MIECs when used in PCFC devices, although the presence of a small amount of protonic conductivity cannot be discounted in these materials.

## Disclosure statement

No potential conflict of interest was reported by the authors.

## Funding

This work was supported by World Premier Research Centre of the Ministry of Education, Culture, Sports, Science and Technology (MEXT).
